# Ecosystem services provided by marine and freshwater phytoplankton

**DOI:** 10.1007/s10750-022-04795-y

**Published:** 2022-01-28

**Authors:** Luigi Naselli-Flores, Judit Padisák

**Affiliations:** 1 Department of Biological, Chemical and Pharmaceutical Sciences and Technologies (STEBICEF), University of Palermo, Via Archirafi, 28, 90123 Palermo, Italy; 2 Research Group of Limnology, Centre for Natural Sciences, University of Pannonia, Egyetem u. 10, Veszprém, 8200 Hungary

**Keywords:** Biosphere’s engineers, Climate regulation, Primary production, Nutrient cycling, Cultural services

## Abstract

Phytoplankton, the ecological group of microalgae adapted to live in apparent suspension in water masses, is much more than an ecosystem’s engineer. In this opinion paper, we use our experience as phytoplankton ecologists to list and highlight the services provided by phytoplankton, trying to demonstrate how their activity is fundamental to regulate and sustain Life on our Planet. Although the number of services produced by phytoplankton can be considered less numerous than that produced by other photosynthetic organisms, the ubiquity of this group of organisms, and their thriving across oceanic ecosystems make it one of the biological engines moving our biosphere. Supporting services provided by phytoplankton include almost half of the global primary and oxygen production. In addition, phytoplankton greatly pushes biogeochemical cycles and nutrient (re)cycling, not only in aquatic ecosystems but also in terrestrial ones. In addition, it significantly contributes to climate regulation (regulating services), supplies food, fuels, active ingredients and drugs, and genetic resources (provisioning services), has inspired artistic and craft works, mythology, and, of course, science (cultural services), and much more. Therefore, phytoplankton should be considered in all respects a true biosphere’s engineer.

## Introduction

It was probably Alexander von Humboldt, one of the most influential scientists ever, the first who, at the end of the eighteenth century, already noted that the human-induced alteration of the environment (the words “ecology” and “ecosystems” did not exist yet), and in particular the extensive transformation of forested areas in agriculture lands, negatively impacted human well-being (Wulf, 2015). However, almost two centuries passed before this concept has started attracting scientific interest with a paper published in 1977 on Science (Westman, 1977). This can be considered the first attempt to find ways to establish public awareness on the fact that environmental pollution leads to changes in the functioning of ecosystems, which are detrimental to human welfare and health (Westman, 1977). In particular, the paper highlights the dependence of humanity on the services provided by Nature and on the role exerted by biodiversity on maintaining “the things that matter to people” (Bekessy et al., 2018).

Since then, ecosystem services have achieved a global interest, especially after Costanza et al. (1997), to reinforce the concept and make it easily understandable to the public, provided a monetary evaluation of the services offered by Nature. This evaluation, updated in 2014 (Costanza et al., 2014), further stimulated the interest in the topic, showing that the capital provided per year by ecosystem services is largely greater than the global GDP. As reviewed by Schröter et al. (2014), the ecosystem services concept has triggered over time several debates, collecting both critiques and counter-arguments. One of the most prominent topics of discussion is centered on the monetary evaluation of ecosystems, which is perceived as an anthropocentric position rather than as a biocentric reasoning addressed at underlining the intrinsic values of Nature. According to this view, the attribution of an economic value to Nature can fuel a market-driven view of the biosphere and the general perception that ecosystems’ integrity is important just because it provides goods of economic value to humanity. Counter-arguments to this view consider that biocentric and anthropocentric positions are not contrastable and that the ecosystem services concept bundles these positions to achieve the protection and the sustainable use of ecosystems in an anthropocentrically dominated world (Luck et al., 2012). In other words, if we want our politicians and policy makers realistically perceive the problem of maintaining ecosystems’ integrity to protect human well-being, we need to offer them a point of view and some reasons that they can not only understand, but also use to include and justify in their agenda ecosystem management approaches.

Ecosystem services concept was also adopted by United Nations which promoted a four-year study mainly addressed to policymakers: the Millennium Ecosystem Assessment (MEA, 2003, 2005). This was followed by a second initiative, undertaken by UNEP and named The Economics of Ecosystems and Biodiversity (TEEB Foundations, 2010). Both initiatives were addressed at clearing the concept that the well-being of humanity depends on the integrity of the services that ecosystems provide to us (La Notte et al., 2017) and at creating a bridge among scientists, managers, politicians, and stakeholders. A further notion recently introduced in the debate about ecosystem services is that of nature’s contribution to people (NCP). According to Díaz et al. (2018), NCP collects both positive and negative contributions of nature to people’s quality of life. This often depends on the perspective from which a contribution is observed: deforestation can contribute economic benefits since it provides wood resources and pasture lands but at the same time is detrimental for local population relying on forest resources for their daily life up to the point that, historically, they had to abandon their original settlements.

However, ecosystems would not exist without their biological components and without the complex network of biological interactions that allow sunlight energy to flow and matter to circulate through biogeochemical cycles within the system itself. In almost all ecosystems, the starting point of their functioning is represented by the conversion of sunlight energy into chemical energy through photosynthesis. Almost all the heterotrophic communities (with the exception of those supported by chemoautotrophs as in the oceanic hydrothermal vents), including the human community, are bottom-up regulated by photosynthetic organisms. Therefore, algae (including cyanobacteria) and plants, the two major groups of photosynthetic organisms, are directly or indirectly key elements in the provision of services in any kind of ecosystem, both terrestrial and aquatic. Although plants are often easily recognized as providers of fundamental services (e.g., food and oxygen supply, soil formation and stabilization, biogeochemical cycles promoters, climate regulators, and so on), algae are not for a variety of reasons. In fact, the benefits they offer are often associated with ecosystems, like oceans and lakes rather than to the algal community itself. One of the reasons, if not the most important, is probably the microscopic size of the majority of algae which perform these functions (and produce their services) while remaining invisible to most observers. Among microscopic algae, phytoplankton is the ecological group of organisms most “performing” in terms of regulating the functions (and thus the services provided) of not only aquatic ecosystems but of the entire biosphere. In spite of this, a few papers exist assessing and analyzing the role of phytoplankton as provider of ecosystem services (e.g., Acevedo-Trejos et al., 2018; Tweddle et al., 2018), although several scientific articles generically report such role as important.

In this opinion paper, we use our experience as phytoplankton ecologists to list and highlight the services provided by phytoplankton, trying to demonstrate how their activity as biosphere’s engineers is fundamental to regulate and sustain Life on our Planet.

## Ecosystem services provided by phytoplankton: classification and some adjustments

The Millennium Ecosystem Assessment (MEA, 2003, 2005) identified about 30 ecosystem services and categorized them into four main groups:

- *Supporting services* This group includes all the services that are instrumental for the functioning of ecosystems and that thus allow the release of all the other services provided by ecosystems (e.g., oxygen production through photosynthesis, primary production, nutrient cycling). Unlike other categories of services, they generally occur over a long period of time.
- *Regulating services* These include the benefits deriving from the regulation of ecosystem processes (e.g., climate regulation, water depuration).
- *Provisioning services* All the products acquired from ecosystems are grouped in this category: e.g., food, fuels, active ingredients and drugs, genetic resources.
- *Cultural services* This group include all the non-material benefits that people receive from ecosystems as spiritual and esthetic experiences, cognitive development, recreational activities.

Phytoplankton, an ecological group of unicellular or colonial photosynthetic organisms adapted to live in apparent suspension in water masses (Reynolds, 2006), is a provider of many of the aforementioned services, in all the identified categories, to the ecosystems in which it lives and also to humankind.

### Supporting services

#### Primary production: oxygen and biomass

**Fig. 1:**
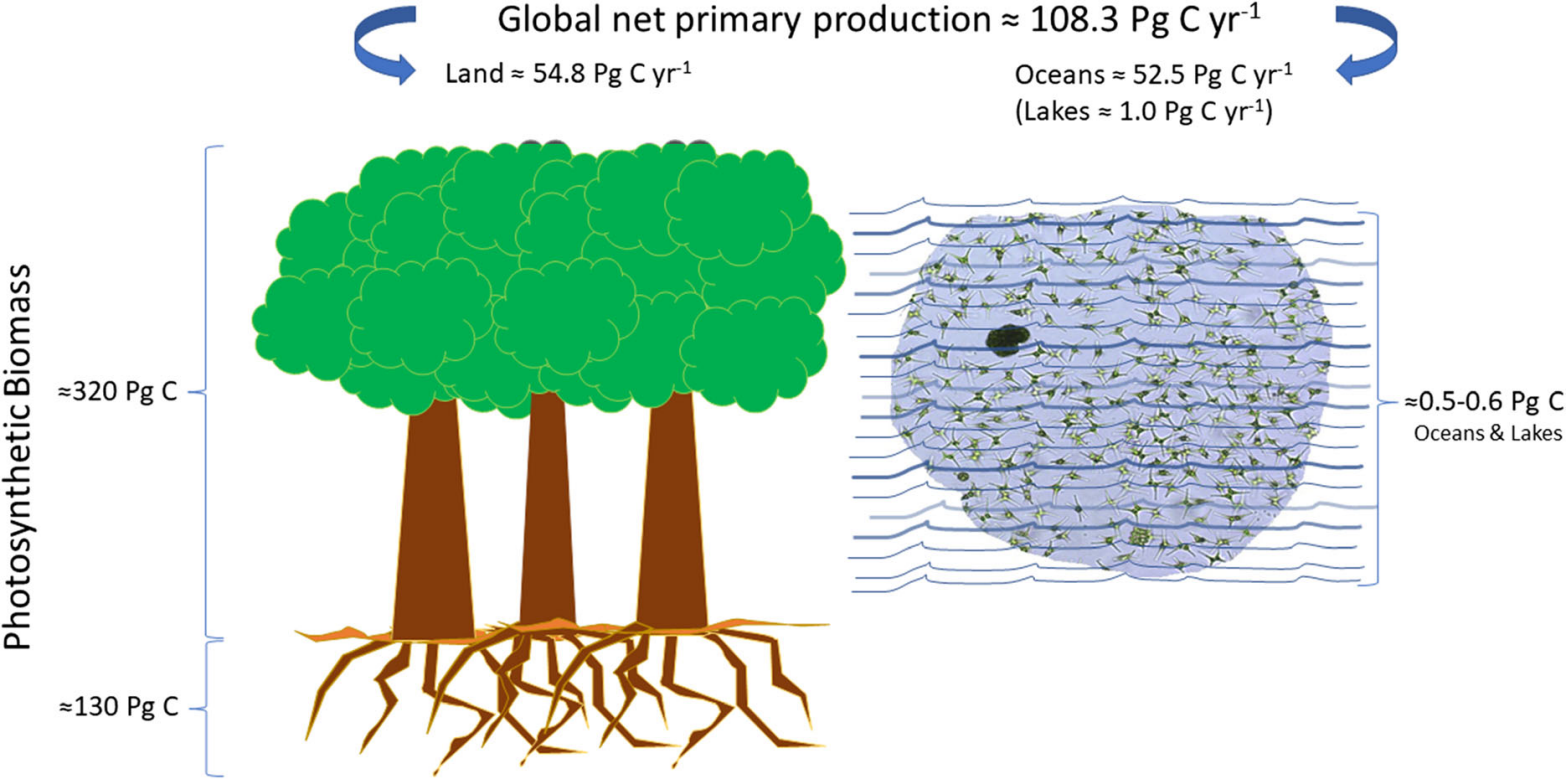
Different contributions of terrestrial vegetation and phytoplankton to global net primary production

About half (49%) of the global net primary production (≈108 Pg C year^−1^) and of atmospheric oxygen come from the photosynthetic activity of phytoplankton (Field et al., 1998; Friend et al., 2009; Lewis Jr, 2011), although, due to a turnover occurring over fast timescales of days, its standing stock represents only < 1% of the global photosynthetic biomass (Sigman & Hain, 2012; Bar-On et al., 2018). Global phytoplankton standing stock in lakes represents a minor percentage (≈ 0.01 Pg C) of that in the oceans and its net primary production has been estimated at 1 Pg C year^−1^, i.e., about 2% of that provided by marine phytoplankton. This value is not negligible considering that global lakes’ surface covers less than 1% of Earth’s surface (Lewis Jr, 2011). Moreover, phytoplankton-fixed carbon almost entirely enters food webs, efficiently promoting energy fluxes and nutrient recycling: two fundamental processes for ecosystems functioning, which can apparently delay but do not suppress the process of carbon sequestration exerted by phytoplankton on a global scale. Conversely, land plants produce huge mass of supportive woody tissue and an extensive root network where most of the fixed carbon is conveyed and made unavailable for a long time (Fig. 1). According to Bar-On & Milo (2019), considering only leaf mass would reduce total land plant biomass (≈450 Pg C) by 30-fold.

We could argue that the relative contribution of phytoplankton to global productivity has probably increased in recent years due to the loss of large forested areas. A large part of Amazon rainforest, for a long time considered the green lung of the planet, is now emitting more CO_2_ than able to absorb due to climate change but also to deliberate fires aimed at clearing land for beef and soy production (Gatti et al., 2021). Clearing rainforest to create more space to agriculture and livestock has been politically and explicitly sustained (Thomaz et al., 2020), probably because of a blind and opportunistic interpretation of the monetary value to be attributed to ecosystems (e.g., a “lucrative” agroecosystem *vs* an “unproductive” rainforest) and without any consideration of Nature’s contribution to people (Díaz et al., 2018). Moreover, deliberate, fraudulent fires and climate change are also plaguing Mediterranean forests and maquis worldwide, also altering the CO_2_ balance in these ecosystems (Hanan et al., 2021; Li et al., 2021).

Due to its productivity both in terms of biomass and oxygen, phytoplankton has been proposed as a first candidate for the establishment of life support systems in space exploration (Wheeler, 2010) and for initiating the “terraforming” process on Mars (Wentz, 2015).

Food production is considered a *provisioning service* since only the contribution to direct human utilization of ecosystem “products” is considered by the MEA. But in the case of phytoplankton some overlap between primary production (supporting service) and food production (provisioning service) exists. Phytoplankton primary production fuels food webs in the majority of aquatic ecosystems, thus supporting fisheries (including aquaculture) worldwide and allowing the role of human food provider offered by aquatic ecosystems. Large marine animals (both fish and mammals) also rely on the food provided by the quite short, and therefore quite efficient food chains as those formed by, e.g., phytoplankton → zooplankton (krill) → great whales (Hill et al., 2006) or in inland saline lakes by phytoplankton → lesser flamingos (Krienitz & Kotut, 2006). Whales can be considered “ecosystem engineers” (see below the paragraph on climate control) and while directly or indirectly being fed by phytoplankton, they return to phytoplankton large nutrient subsidies (especially limiting nutrients as Fe or N) both on a local scale along ocean’s depth (whale pump) when they feed in the deep part of the ocean and release fecal plumes at the surface, and on a global scale (great whale conveyor belt) when they migrate from the Northern, nutrient-rich feeding-grounds to the tropical and subtropical, nutrient-poor breeding grounds (Roman et al., 2014).

Terrestrial ecosystems also benefit from phytoplankton primary production since organic inputs from the ocean (through seabirds’ excrements, “guano”, as an example) can support high productivity on small islands, and coastal areas (Polis & Hurd, 1996). Moreover, the guano trade supplied huge amounts of fertilizers to agroecosystems worldwide in the nineteenth and early twentieth centuries (Cushman, 2013). Marine-derived nitrogen, phosphorus, and other micronutrients from nutrient-rich aquatic ecosystems are incorporated by anadromous fish like salmons into their body tissues (Merz & Moyle, 2006). When spawning salmons return to the streams where they were born, these tissues provide a dominant nutrient subsidy to terrestrial forests and enhance their biodiversity (e.g., Hocking & Reimchen, 2002; Wagner & Reynolds, 2019). In fact, salmons are preyed by bears, wolves, eagles, and their carcasses enter the terrestrial detritus chain, delivering to forests a legacy of keystone nutrients originally fixed into organic matter by phytoplankton (Hilderbrand et al., 1999).

#### Biogeochemical cycles and nutrient recycling

**Fig. 2:**
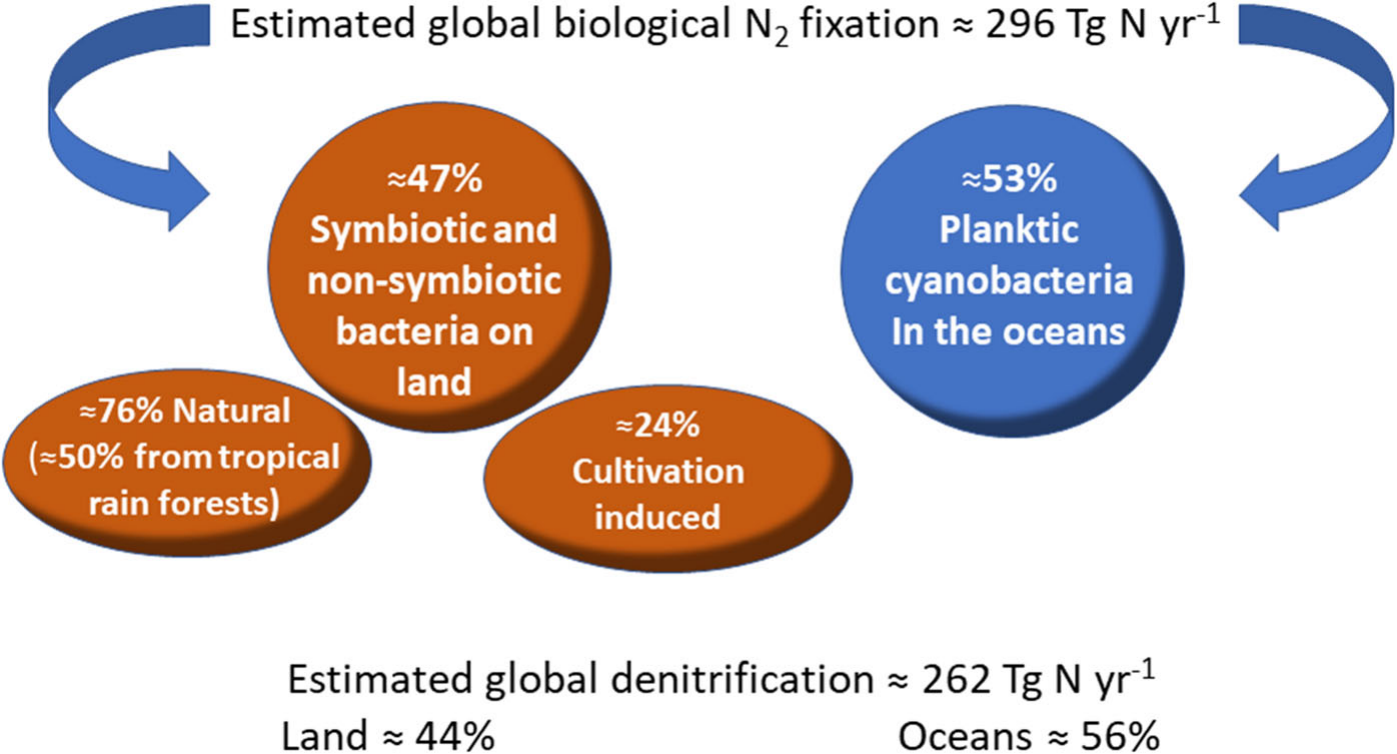
Different contributions of terrestrial bacteria and marine planktic cyanobacteria to global biological nitrogen fixation

Phytoplankton species have an important role in the biogeochemical cycles of all the inorganic elements necessary to support life. Forming the base of aquatic food webs, and also contributing to those of terrestrial ecosystems, phytoplankton provides a fundamental supporting service to the biosphere: nutrient (re-)cycling and redistribution. Phytoplankton establishes microbial interactions with viruses, archaea, bacteria, and fungi. This constitutes one of the most important inter-organism associations in the biosphere that influence the global cycling of micro- and macronutrients (Kamalananthan et al., 2021). Actually, phytoplankton produces the oxygen used by microorganisms for the aerobic decomposition of organic matter, and supports nutrient recycling at different temporal scales, from very short ones, through the so-called microbial loop (Azam et al., 1983) and myco-loop (Kagami et al., 2007), to much longer ones when it enters the phytoplankton → zooplankton → nekton food chain and the nutrients are released as fecal plumes and urine (Roman et al., 2014). Moreover, diazotrophic cyanobacteria, a common and often abundant group of phytoplankton, can fix atmospheric nitrogen and make it available to non-diazotroph phytoplankton and to secondary producers as organic matter. Some widespread planktic diatoms (e.g., *Chaetoceros*, *Rhizosolenia*) can also contain symbiotic diazotrophic cyanobacteria (*Richelia intracellularis* C. H. Ostenfeld ex J. Schmidt, *Calothrix rhizosoleniae* Lemmerman) which provide their host (and their consumers) with bioavailable nitrogen in oligotrophic environments (Jabir et al., 2013). On a global scale, cyanobacterial diazotrophy is the largest source of the newly fixed nitrogen in the oceans. The process counterbalances losses due to denitrification and anaerobic ammonium oxidation (Nieder & Benbi, 2008), fuelling, in some oligotrophic oceanic regions, up to 50% of the total production. Global marine N_2_ fixation has been estimated to range between 100 and 200 Tg N year^−1^ (Fig. 2), with the genus *Trichodesmium* contributing to ≈43% of the total, unicellular diazotrophs to ≈49% and diatom-diazotroph associations to ≈8% (Monteiro et al., 2010; Bergman et al., 2013).

Most plants in aquatic environments prefer or tolerate  deviations of about ± 2.5 from the pH neutral conditions. If the habitat offers harsher conditions, only some specially adapted phytoplankton species are able to act as primary producers (Padisák & Naselli-Flores, 2021). This way, these species expand the inhabitable, autotrophic locations of the Earth.

#### Sediment formation

In a few cases, when referring to supporting services as listed by MEA, some adjustments can be necessary. As an example, phytoplankton does not contribute to “soil formation” listed by MEA as one of the most important supporting services, which indirectly affects people through food production (provisioning service). However, this group of organisms, when sinking to the bottom of the water bodies, participate to sediment formation, sustain benthic communities, and eventually become part of the sedimentary archives which can provide useful information to paleoceanographers and paleolimnologists, when investigating past environmental conditions of our planet, thus providing an important *cultural service* and also serving as predictive tool for reconstruction of past ecological status of lakes and for assessing consequences of the ongoing climate change (e.g., Buczkó et al., 2009). In addition, calcifying phytoplankton and diatoms have been participating to sediment formation since millions of years and have contributed to the formation of sedimentary rocks like limestone and diatomite, widely used by humans as building material for centuries. As an example, high abundance of coccolithophores formed the widespread chalk deposits like the renowned cliffs of Dover in UK (Püttmann & Mutterlose, 2021). Limestone sedimentary rocks made of calcifying phytoplankton remains may have contributed to the Giza Pyramids in Egypt or to St. Peter cathedral in Rome, whereas diatoms contributed to the light-weight building material (diatomite rock) for the dome of Hagia Sophia in Turkey (see https://www.sandatlas.org/diatomaceous-earth/#1); therefore, house and monument building in the world may have largely benefited from sediment formation promoted by phytoplankton. Diatomite, and the powder derived from it, has several industrial uses: Alfred Nobel employed it as a stabilizer for nitro-glycerine in the production of dynamite (Wisniak, 2008); it is widely used in beer production for filtration and transparency enhancement and even secondary use of diatomite from brewery are offered (e.g., Goulart et al., 2011; Dessalew et al., 2017). Sediment formation by coccolithophores and planktic diatoms has been ongoing, even though the benefits from the present process will be available in the next million years.

### Regulating services

#### Air quality maintenance and climate regulation

Being responsible of about half of the Earth’s oxygen production, phytoplankton represents an important “green lung” for our planet and thus largely contributes to provide ecosystems with one of their regulating services: air quality maintenance. Moreover, the photosynthetic process underpinning oxygen production is based on the acquisition of CO_2_ as a carbon source for biomass production. With regard to phytoplankton, part of this carbon is used by calcifying phytoplankton species (e.g., coccolithophores) belonging to the division Haptophyta to produce their characteristic scales (coccoliths) made of calcium carbonate (CaCO_3_). Coccoliths cover the cell surface in the form of a spherical coating called coccosphere. These algae have been an important part of marine phytoplankton assemblages since the Jurassic (Bown et al., 2004). In the modern ocean, coccolithophores are a key phytoplankton group and represent up to 20% of marine primary production (Poulton et al., 2017). One of the most common species of this group, *Emiliania huxleyi* (Lohmann) Hay & Mohler, can form widespread blooms worldwide. These, although characterized by relatively high numbers of cells, generally show chlorophyll *a* concentration lower than 2 mg Chl *a* m^−3^, due to the low chlorophyll content of their cells (Hopkins et al., 2015). Once these organisms die, they sink and their calcium carbonate scales are partly stored in the geological archives (Westbroek et al., 1993). Most of the carbon buried in marine sediments as CaCO_3_ has a biogenic origin (Broecker & Clark, 2009) and about 60% of the total carbonate flux is due to coccolithophores (Haidar et al., 2000). Therefore, this phytoplankton group has a major influence on the marine carbon cycle and on the inorganic carbon pump, significantly contributing to the sequestration of large amount of CO_2_ from the atmosphere and providing an important regulating effect not only to the ecosystems where they thrive but to the entire biosphere (Haunost et al., 2021). Altogether, a fraction ranging between 20 and 35% of global annual CO_2_ emissions are directly sequestered by phytoplankton (Khatiwala et al., 2009). On the whole, the amount of CO_2_ captured yearly by phytoplankton has been estimated to be equivalent to that captured by 1.7 trillion trees, i.e., four Amazon forests’ worth (Chami et al., 2019). In freshwater lakes with the most frequent slightly alkaline pH, free CO_2_ is not available in necessary amounts. In such lakes phytoplankton species possessing the enzyme carbon anhydrase take up HCO_3_^−^ and then generate partly CO_2_, partly CO_3_^2−^. The latter is released by the cells and with the dissolved Ca^2+^ in the surrounding water forms fast settling CaCO_3_ precipitates. The process is called biogenic calcite precipitation during which PO_4_^3−^, the typical limiting nutrient in such lakes, co-sediments with the biogenic calcite. This process used to be considered as a major natural biogeochemical process to mitigate anthropogenic eutrophication (Koschel et al., 1987) even though the P sedimented this way may result, after re-dissolving of co-precipitates, in sudden internal P-loadings and subsequent proliferation of diazotrophic cyanobacteria especially when “assisted by” the extremities of the climate change (Kasprzak et al., 2017; Selmeczy et al., 2019). In hardwater lakes the volvocalean *Phacotus lenticularis* (Ehrenberg) Diesing with its loricae may contribute significantly to calcite precipitation and therefore to biogeochemical carbon cycling (Lenz et al., 2018).

An indirect contribution provided by phytoplankton to carbon sequestration is linked to its role as primary producer. All the marine animals that directly or indirectly rely on phytoplankton for their food, sooner or later die and their carcasses sink to the deep removing significant amounts of carbon from the atmosphere. Whales, in particular, live for many decades and the carbon stored in their body will remain out of the atmosphere for the animal’s life. It has been estimated that whale falls alone transfer 190,000 tons C year^−1^ from the atmosphere to the ocean beds, a value which would almost double by stopping whale hunting (Pershing et al., 2010).

In addition, phytoplankton species (e.g., *Chrysochromulina* spp., *Gyrodinium flagellare* Schiller, *Emiliania huxleyi* and many others) produce dimethylsulfoniopropionate (DMSP), an important metabolite in the marine sulfur cycle, as an osmolyte and cryoprotectant (Scarratt et al., 2002). When released into the water, DMSP is transformed into the volatile dymethylsulfide (DMS) which represents the principal source of sulfate aerosols in the troposphere. Sulfate aerosols have an important role in the formation of cloud condensation nuclei and in the formation, persistence, and albedo of clouds. Accordingly, Charlson et al. (1987), in one of the first papers dealing with climate change, proposed the possibility of a biological control of climate by influencing phytoplankton DMSP production.

#### Biological control

Among phytoplankton species significant variability exist in their growing rates (Reynolds, 2006). As an example, green algae and diatoms can grow much faster than toxin-producing cyanobacteria under adequate light and nutrient conditions (Naselli-Flores & Barone, 2011; but see also Lürling et al., 2013). Therefore, non-toxic species could outcompete toxic ones and exert a kind of biotic resistance against their spreading if the environmental conditions were suitable. According to MEA (2003), “ecosystem changes affect the prevalence of crop and livestock pests and diseases”. As regards the aquatic ecosystems, environmental changes as those caused by eutrophication, salinization, acidification, and climate change have impaired the biotic resistance offered by non-toxic phytoplankton species, favoring in the last decades the significant increase of harmful algal blooms worldwide (Jeppesen et al., 2015; Gobler, 2020). Ecosystem changes, as those that occurred to aquatic ecosystems in the last 50 years, have impaired one of the ecosystem services potentially provided by phytoplankton, i.e., the biological control against nuisance species. Restoring the environmental conditions that allow non-toxic phytoplankton species to dominate would increase the positive effect they exert against harmful algal blooms (Naselli-Flores, 2014).

### Provisioning services

#### Food, bioactive compounds, “green chemistry”

Phytoplankton direct use as food by human populations has occurred for centuries, especially in Africa and Asia. Due to their high content in proteins and carbohydrates, some cyanobacteria like *Limnospira* (synonims: *Spirulina*, *Arthrospira*) and *Aphanizomenon* have been harvested to provide food for thousands of years (Spolaore et al., 2006). It was, however, in the early 1950’s, due to the human demographic increase, that the systematic search on phytoplankton biomass as a new food source started (Becker, 2004). Contemporary, several studies started identifying microalgae as a source of biologically active substances (Borowitzka, 1995). Since then, it has become clear that, as the majority of photosynthetic organisms, phytoplankton produces a vast array of biologically active metabolites, especially regarding its biochemical diversity. Among these, phytoplankton-derived fatty acids, amino acids, carotenoids, vitamins, enzymes, sterols, inorganic and organic minerals, chlorophyll, and trace elements (Napiórkowska-Krzebietke, 2017) can be commercially exploited and have stimulated industrial interests. In fact, these bioactive compounds can find several applications in, e.g., pharmaceuticals (e.g., Casagrande do Nascimento et al., 2019; Ochoa-Méndez et al., 2016) and nutraceuticals production (Fields et al., 2020), production of vitamins, food additives and animal food production (Spolaore et al., 2006), and cosmetics (Lupette & Maréchal, 2018; Jacob-Lopes et al., 2019). Among phytoplankton groups known to produce secondary bioactive metabolites, cyanobacteria are one of the most studied. Although these organisms are mainly known to produce toxins which may cause a variety of problems to human and environmental health (Chorus & Welker, 2021), some studies have shown that these secondary metabolites can be helpful to human health because of their immuno-enhancer and anticancer property (Jensen et al., 2001; Qamar et al., 2021). Moreover, the potential allochemical role of these substances has been proposed as a source of natural alternatives to synthetic pesticides (Berry et al., 2008). Last, the need to find an alternative to fossil fuels in the production of plastics is promoting new research in the field of “green chemistry” and the potential of phytoplankton species in the production of bioplastics and textiles has been receiving increasing interest (Cinar et al., 2020).

#### Fuel production

Although still controversial, it seems that fossil fuel on our planet have a biogenic origin and they come from organic matter produced, among others, by phytoplankton and accumulated on the ocean floor in a process that started in the Mesozoic age (252–266 million years ago) and took millions of years to form the current deposits (Walters, 2006). Due to the long time required for deposits’ formation and for the changes in the conditions that allowed their formation, fossil fuels are considered a not-renewable resource even though their burning still represents the main energy supply for humanity and the main engine of world economy. Our energetic dependency from fossil fuels burning has caused the fast re-emission in the atmosphere of huge amount of CO_2_ photosynthetically sequestered during millions of years and triggered climate and global change. This unsustainable consumption of fossil fuels could be partially counterbalanced by biofuels production from microalgae (Pienkos & Darzins, 2009; Vanthoor-Koopmans et al., 2013). Several phytoplankton species, both freshwater and marine (e.g., *Botryococcus braunii* Kützing, *Chlamydomonas reinhardtii* P.A. Dangeard, *Chlorella* spp., *Dunaliella* spp., *Prymnesium parvum* Carter, *Skeletonema costatutm* (Gréville) Cleve, *Picochlorum* spp.), can produce, in a fast way, large amounts of hydrocarbons, especially lipids, which are suitable for biodiesel production (Razeghifard, 2013; Mucko et al., 2020). In this respect, we have to keep in mind that biofuel production from land plants (e.g., soybean, oil palm, sugarcane, wheat, maize) causes changes in the land use and a loss of biodiversity (Tudge et al., 2021). Conversely, microalgae can be grown in a more efficient and sustainable way since they do not require fertile land to grow (Yamamoto et al., 2016).

#### Genetic resources, basic research

Considering all the bioactive molecules they produce and the biotechnological applications which could be provided by its species and strains, phytoplankton represents an important genetic resource. Many species in this group have shown potential for biotechnological manipulation and genetic modification of their metabolic pathways aimed at improving their productivity as a source of food, bioactive compounds and fuels (Singh et al., 2011; Tanabe et al., 2015).

Among phytoplankton, species belonging to the genera *Chlorella*, *Scenedesmus*/*Desmodesmus,* and *Chlamydomonas* were the first microorganisms used for biochemical and physiological analyses of the cell cycle more than 60 years ago (Borowitzka et al., 2016). In particular, *Chlamydomonas reinhardtii* P. A. Dangeard is considered a model organism that largely has contributed to advance human knowledge on cell biology, physiology, and genetics of plants and animals. Most of the knowledge on the relationships between genes, their encoded proteins, and the functional roles they exert, without mentioning cell evolution and phylogeny, photosynthesis, respiration, calcium metabolism, axonemal structure and function, and the evolution of vision has been achieved by using *C. reinhardtii* as a model (Hippler, 2017). Another organism, the *Synechocystis* PCC 6803 small, coccal cyanobacterium strain has more than 40,000 items at the Google Scholar.

### Cultural services

#### Myths, legends, transcendent beliefs, arts, crafts and education, tourism

Some planktic microalgae produce pigments (e.g., astaxanthin, phycoerythrin) that, in case of blooms, produce a red colouration of the surface waters. This phenomenon is at the origin of several myths and religious beliefs and also has inspired artists over the centuries.

According to Fogg (2002), the first plague of Egypt (the Plague of Blood, Exodus 7: 14–25), when the River Nile water turned to, the fish died and the people could not drink from the Nile, was attributable to a toxic dinoflagellate bloom. The Red Sea owes its name to the huge blooms of the cyanobacterium *Trichodesmium erythraeum* Ehrenberg ex Gomont (Capone et al., 1997). These blooms likely originated the Biblical episode in which Moses and the Israelites pass through the Red Sea while the Egyptians army is destroyed. The episode inspired the early-baroque painter Antonio Tempesta who rendered it in an oil-painting on Italian red marble (https://www.youtube.com/watch?v=kBIzRpf893k). Moreover, reddening of ocean’s surface intrigued naturalists like Darwin and sailors like captain James Cook who wrote about this phenomenon in their travel diaries (Capone et al., 1997). In addition, in Lake Murten (Switzerland) the freshwater, red-pigmented cyanobacterium *Planktothrix rubescens* (de Candolle ex Gomont) Anagnostidis et Komárek was interpreted as the blood of Burgundian soldiers whose bodies were thrown in the lake after the siege of Murten in 1476, feeding a long-lasting myth (Walsby et al., 2006). Therefore, the common German name of *P. rubescens* is “Blutalga”. *P. rubescens* blooms may have also contributed to create the legend of the Red Cock in the German Brandenburg region. According to this legend, the Red Cock lives on the deep bottom of Lake Stechlin. From time to time it appears at the surface, red and angry, and beats the lake with its wings until it foams and surges, causing deaths among humans (Padisák et al., 2010a).

Not only the colors, but also the striking variability of phytoplankton shapes has been an inspiration for science and art. In the early twentieth century, the German biologist Ernst Haeckel published his multi-volume series *Kunstformen der Natur* (Artforms in Nature) with several plates containing drawings of phytoplankton species. By browsing these drawings clearly appears how much this work influenced the Art Nouveau design and architecture (Willmann & Voss, 2017).

Phoenix is the name of many burial services all over the world as symbol of reincarnation or immortal life. It originates from a Greek mythology but parallels many similar legends all around the world (Blake, 1964; Gerlach, 1998). The Phoenix is a bird, falling into the sea and then emerges again shining lively. Indeed, if a bird takes up from the coastal region with blooms of bioluminescent dinoflagellate species (e.g., *Alexandrium, Lingulodinium, Protoceratium, Pyrocystis, Noctiluca*) it is shining as the surface of the bird is covered by dinoflagellates and the flying mechanics triggers the luciferase-luciferin reaction resulting in the “firebird” impression that inspired even musicians, being the widely known Igor Stravinsky (https://en.wikipedia.org/wiki/The_Firebird). The Phoenix legends are not exclusive for people living in coastal regions. Lesser flamingo feeding on the pink *Limnospira* in African saline lakes is also considered as firebird, symbol of immortality and appears in various legends (see in detail in Krienitz, 2018).

The beautiful shapes of phytoplankton organisms have been inspiring jewel makers since a couple of years. By searching the internet using “phytoplankton jewelery” as a keyword (https://www.google.com/search?q=phytoplankton+jewellery&source=lnms&tbm=isch&sa=X&ved=2ahUKEwj9wv-Vi-3yAhWGOOwKHRQkA_wQ_AUoAXoECAEQAw&biw=1366&bih=625), many different species of planktic microalgae can be found that have been reproduced in bracelets, pendants and earrings.

#### Model organisms for plant science and ecology/evolution

Phytoplankton species are easily cultivated under lab conditions and culture collections of hundreds of species are existing since decades. This has contributed to significantly increase scientific knowledge in population and community ecology since many species with different ecological/physiological performances can be available in large numbers and successfully used to investigate the role of biological interactions as competition and predation under different environmental conditions (Reynolds, 2006) and testing general ecological concepts like the Intermediate Disturbance Hypothesis (Flöder & Sommer, 1999). Phytoplankton has been frequently used as a model to investigate, among others, population growth and assembly rules (e.g., Reynolds et al., 2002; Padisák et al., 2009; Rojo, 2021), adaptations to extreme environmental conditions (e.g., Padisák & Naselli-Flores, 2021), relationships between predators and preys (e.g., Harvey & Menden-Deuer, 2012), and understanding the pathways to adaptation for living in suspension (Naselli-Flores et al., 2021).

Last but not least, phytoplankton is also a reference assemblage in biomonitoring of aquatic ecosystems since the organisms belonging to this group are easily available in large numbers, show an amazing diversity in terms of adaptability to environmental conditions, and are characterised by fast growth rates which make them suitable as an early warning indicator of ecosystem changes (Salmaso et al., 2012). Especially for lakes, but also for rivers phytoplankton serves as one of the biological qualification elements to assess ecological quality of surface waters according to the guidelines of the EU Water Framework Directive (e.g., Birk et al., 2012).

Though not often, but phytoplankton was used in natural science education. The recently passed, famous phytoplankton ecologist Colin S. Reynolds suggested to teach food web structure and function using Lego bricks for elementary school children (Reynolds, 1994). Another example is the paper by Ebert & Müller (2012) teaching about importance of form and function in context of particle settling in fluid media.

#### Tourism

Relationship between tourism and phytoplankton is indirect and is largely associated with negative or even harmful experiences (the beach is like a pea-soup because of eutrophication, cyanobacterial scums cover the surface, toxic events). However, in some cases, just particular phytoplankton species invisible to the naked eye provide the base of mass tourism. Such is the case in the African “flamingo lakes” attracting millions of tourists to see the crowds of these beautiful pink birds. Lesser flamingo, *Phoeniconaias minor* (É. Geoffroy Saint-Hilaire, 1798), grazes directly on the large, filamentous, spiraling cyanobacterium, *Limnospira fusiformis* (Voronichin) Nowicka-Krawczyk, Mühlsteinová & Hauer. “Flamingo lakes” lakes are saline selecting for only one or some phytoplankton species that can cope with the prevailing environmental constraints but these species can sustain their populations over years (Padisák & Naselli-Flores, 2021). If the *Limnospira* is replaced by a species (e.g., *Picocystis salinarum* R. A. Lewin; Pálmai et al., 2020) falling below the grazeability threshold of the lesser flamingo, the birds abandon the lake that results in regional GDP loss and economic crisis.

## Phytoplankton biodiversity contribution to ecosystem services

**Fig. 3:**
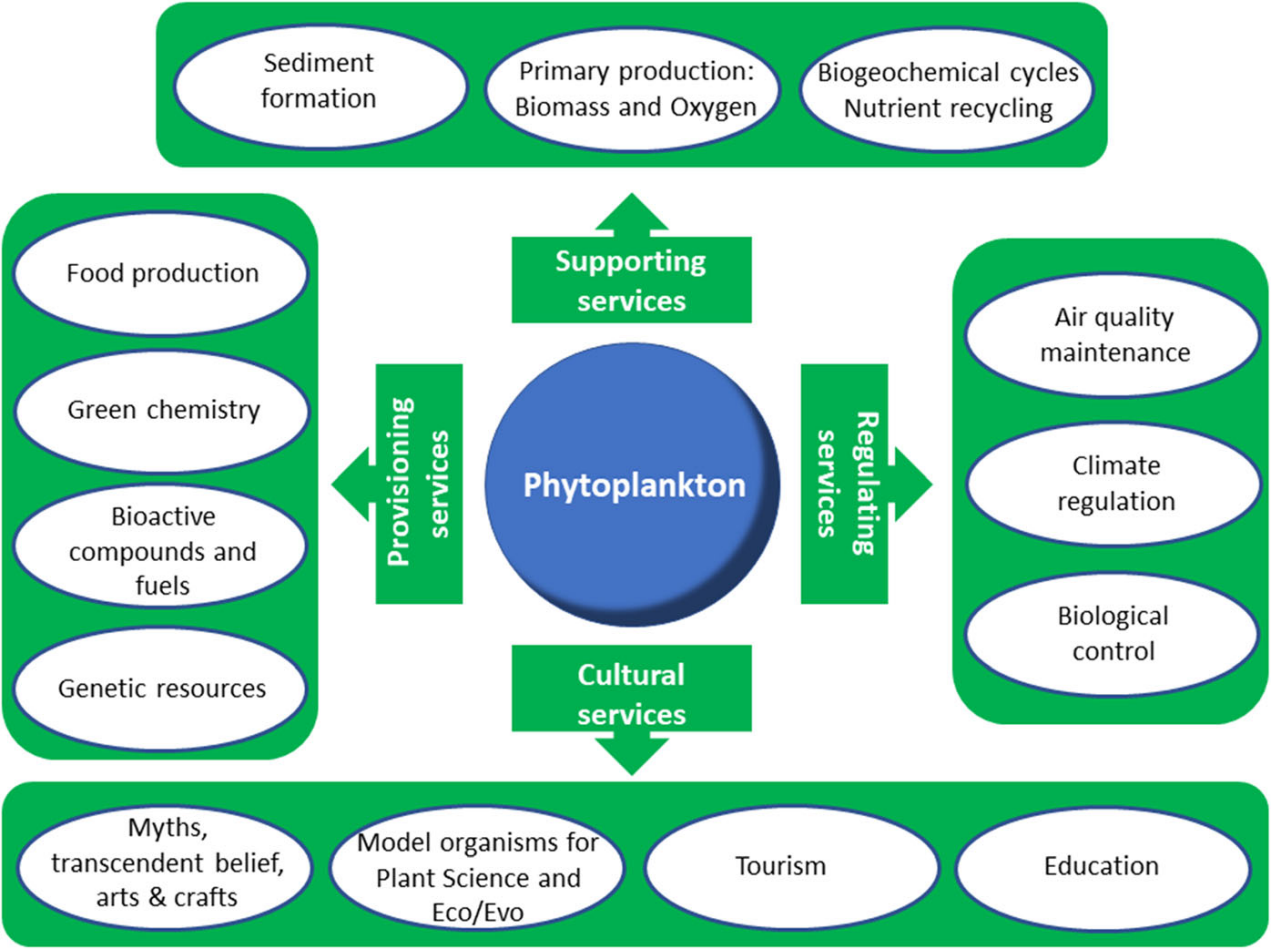
Schematic block diagram showing the ecosystems services categories and the identified ecosystem services provided by phytoplankton

Although at a local scale phytoplankton may support fewer ecosystem services compared to macrophytes (Janssen et al., 2021; Thomaz, 2021), we could identify about 20 different ecosystem services globally provided by phytoplankton (Fig. 3). Among these, the supporting services significantly have been sustaining the functioning of large part of the biosphere since millions of years. As shown by Ptacnik et al. (2008), the efficiency of phytoplankton resource use, i.e., carbon fixation, is directly linked to its diversity which also strongly contributes to the global stability of aquatic ecosystems and enhances the role of phytoplankton as provider of regulating services to the biosphere. These tasks have been achieved thanks to the biological diversity shown by this group of organisms in its broadest sense, i.e., in terms of species, genes and functional diversity (Reynolds et al., 2002; Padisák et al., 2009; Acevedo-Trejos et al., 2018; Kruk et al., 2021; Abonyi et al., 2021).

Current estimates suggest that about 10,000 phytoplankton species (equally distributed between marine and freshwater taxa) have been described (Reynolds, 2006). This number most likely underestimates the real number of phytoplankton species since several species are often found to be hidden under one single species name (cryptic species) and/or are not distinguishable by traditional light microscopy (e.g., Moore et al., 2002; Krienitz & Bock, 2012; Komárek, 2018). Although the number of phytoplankton species is much lower than the approx. 308,000 species of vascular plants described up to now (Christenhusz & Byng, 2016), this is a very heterogenous ecological group of photosynthetic organisms that does not collect just a number of distinct taxa of photosynthetic organisms but a wide variety of shape, size, biochemical and phylogenetic affinity. The dimensional range of phytoplankton (1–200 μm) is comparable to the one spanning forest trees and the herbs that grow at their base (0.1–20 m). Moreover, a high intraspecific phenotypic plasticity exists as well as a great interspecific morphological variability. The phyletic divergence of the phytoplankton representatives is yet wider. All these features contribute to the wide diversity that these organisms show in their requirements, dynamics and susceptibility to loss which confer them the possibility to thrive under all the present environmental and hydrodynamic conditions offered by aquatic ecosystems and to provide their services to our planet (Padisák et al., 2010b; Salmaso & Tolotti, 2021; Naselli-Flores et al., 2021).

## Final remarks

Although some negative contributions of phytoplankton to people’s quality of life can be recognized (e.g., blooms of toxic species that impair some provisioning and cultural services), supporting services provided by phytoplankton have sustained the evolution of human species. The technological progress very recently achieved by *Homo sapiens* Linnaeus, 1758 is now impairing, as a boomerang, the equilibria which govern the biosphere functioning as we know it. Greenhouse gases emissions are the main drivers of global change and deeply affect the functioning of aquatic ecosystems through, e.g., (i) acidification and its consequences on microorganisms’ calcification and CO_2_ sequestration; (ii) decrease in oxygen content and its consequences on decomposition and biogeochemical cycles; and (iii) rise in temperature and its consequences on stratification patterns, nutrient recycling, and on the physiology of aquatic primary producers (e.g., Jane et al., 2021 and literature therein; D’Amario et al., 2020; Ripple et al., 2021; Tait et al., 2021). A worsening in several climate-related variables has been recorded by Ripple et al. (2021) in the last 2 years in spite of the slowing down of several impacting human activities due to COVID-19 pandemics. All these changes are severely impacting aquatic ecosystems at different spatial (from watershed to global) and temporal (from transient to chronic) scales and with different intensities (Salmaso & Tolotti, 2021, and literature therein). While transient effects can be compensated at a global scale and can be subjected to human management to reduce their impacts, chronic effects, as the global temperature increase, are destined, without actions or with the present level of actions, to get worse by the end of this century, with the risk of impairing global primary production and biogeochemical cycles in the near future, and ultimately the role of biosphere’s engineers provided by phytoplankton. It is thus of paramount importance to develop immediate mitigation measures addressed at containing the global temperature increase below 1.5 °C. The recent COVID-19 pandemics has shown to the world governments that some solutions cannot be achieved by single nation’s prescriptions but require a tight cooperation and common plans from all the nations since the survivorship and well-being of the entire humanity are involved.

## Data Availability

N.A.
